# *Notes from the Field:* HIV Outbreak During the COVID-19 Pandemic Among Persons Who Inject Drugs — Kanawha County, West Virginia, 2019–2021

**DOI:** 10.15585/mmwr.mm7102a4

**Published:** 2022-01-14

**Authors:** Rebecca B. Hershow, Suzanne Wilson, Robert A. Bonacci, Molly Deutsch-Feldman, Olivia O. Russell, Sherri Young, Shannon McBee, Erica Thomasson, Shawn Balleydier, Miracle Boltz, Vicki Hogan, Amy Atkins, Nancy Worthington, Robert McDonald, Monica Adams, Anne Moorman, Danae Bixler, Stephen Kowalewski, Melinda Salmon, R. Paul McClung, Alexandra M. Oster, Kathryn G. Curran

**Affiliations:** ^1^Epidemic Intelligence Service, CDC; ^2^Division of HIV Prevention, National Center for HIV, Viral Hepatitis, STD, and TB Prevention, CDC; ^3^West Virginia Bureau for Public Health, West Virginia Department of Health and Human Resources; ^4^Division of Tuberculosis Elimination, National Center for HIV, Viral Hepatitis, STD, and TB Prevention, CDC; ^5^DLH Corporation, Atlanta, Georgia; ^6^Kanawha-Charleston Health Department, Charleston, West Virginia; ^7^Division of Overdose Prevention, National Center for Injury Prevention and Control, CDC; ^8^Division of STD Prevention, National Center for HIV, Viral Hepatitis, STD, and TB Prevention, CDC; ^9^Division of Viral Hepatitis, National Center for HIV, Viral Hepatitis, STD, and TB Prevention, CDC.

During October 2019, the West Virginia Bureau for Public Health (WVBPH) noted that an increasing number of persons who inject drugs (PWID) in Kanawha County received a diagnosis of HIV. The number of HIV diagnoses among PWID increased from less than five annually during 2016–2018 to 11 during January–October 2019 (Figure). Kanawha County (with an approximate population of 180,000*) has high rates of opioid use disorder and overdose deaths, which have been increasing since 2016,^†^ and the county is located near Cabell County, which experienced an HIV outbreak among PWID during 2018–2019 (*1*,*2*). In response to the increase in HIV diagnoses among PWID in 2019, WVBPH released a Health Advisory^§^; and WVBPH and Kanawha-Charleston Health Department (KCHD) convened an HIV task force, conducted care coordination meetings, received CDC remote assistance to support response activities, and expanded HIV testing and outreach. 

**FIGURE:**
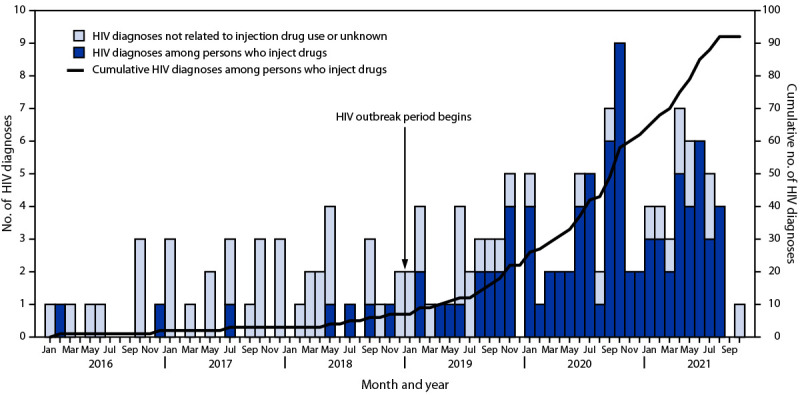
Diagnoses of HIV infection, by injection drug use category — Kanawha County, West Virginia, January 2016–October 2021

After suspension of the KCHD syringe services program (SSP) in March 2018 and a community-based SSP in April 2021 (because of concerns about program administration), a state law^¶^ and a Charleston City Council ordinance** enacted stricter SSP requirements. No new SSPs have opened in Kanawha County since the legislation passed. During 2020–2021, the COVID-19 pandemic affected HIV response activities and in-person services for PWID (e.g., curtailment of partner services,^††^ limitation in outreach testing, and closure of drop-in centers). In April 2021, WVBPH requested partner services surge support, and in May 2021 requested CDC assistance with an HIV outbreak investigation; CDC provided surge and investigation support during April–August 2021. 

An HIV outbreak case was defined as a confirmed HIV diagnosis on or after January 1, 2019 in a PWID living in Kanawha County at the time of diagnosis. Investigators conducted qualitative interviews with 26 PWID and 45 community partners (including service providers),^§§^ and for 65 PWID with HIV, abstracted medical records for 496 health care encounters beginning 1 year before HIV diagnosis through June 18, 2021.^¶¶^ This activity was reviewed by CDC and conducted consistent with applicable federal law and CDC policy.***

As of October 27, 2021, 85 persons met the HIV outbreak case definition; 54 (52%) patients were male, 67 (79%) were aged 20–39 years at diagnosis, and 77 (91%) were non-Hispanic White. Forty patients (47%) had experienced unstable housing during the past year, and 73 (86%) had previous or current hepatitis C infection. Among 80 living persons, 20 (25%) had an HIV care visit during the preceding 90 days,^†††^ and 26 (33%) were virally suppressed based on last test results.^§§§^ Among 25 persons with available HIV molecular sequencing data, 19 (76%) were molecularly clustered (i.e., had an HIV sequence that was closely related to the HIV sequence of one or more other persons), indicating recent HIV transmission. Fifteen (79%) persons were in one molecular cluster, unrelated to the cluster identified during the Cabell County outbreak investigation (*2*).

Interview and medical record data indicated that methamphetamines and heroin were the most frequently injected drugs, and polysubstance use was common (57 [88%] of 65 patients). PWID reported reusing or sharing syringes, mainly because of limited access to sterile syringes after SSP closures. PWID expressed medical mistrust because of experiences of stigma and discrimination in health care settings. Medical record abstraction revealed that HIV screening tests were performed at fewer than one third of health care encounters before diagnosis, and none of the patients had been prescribed preexposure prophylaxis (PrEP). Prescriptions of naloxone for overdose prevention and medications for opioid use disorder were documented at fewer than a quarter of opioid-related health care encounters.^¶¶¶^ Service providers described challenges reaching PWID, including COVID-19 restrictions (e.g., drop-in center closures and outreach activity restrictions) and low SSP access because of some community opposition to evidence-based SSPs and new legislation restricting SSPs.

Recommendations based on investigation findings and HIV surveillance data are guiding response activities.**** WVBPH and KCHD are expanding HIV and hepatitis C testing and PrEP access with partners, training service providers on HIV and stigma reduction, and enhancing care coordination by improving linkage to HIV and substance use services and hiring additional partner services staff members. Stigma and discrimination and low SSP access have posed challenges to engaging PWID in HIV prevention and treatment; these challenges have been exacerbated by the COVID-19 pandemic (*3*). Increasing access to comprehensive harm reduction services (e.g., SSPs) through expansion of mobile services, street outreach, and telehealth encounters led by patient-trusted staff members might improve delivery of important health and social services to PWID (*4*,*5*).

## Footnotes

* https://www.census.gov/quickfacts/kanawhacountywestvirginia (Accessed January 10, 2021).

†
https://dhhr.wv.gov/office-of-drug-control-policy/datadashboard/Pages/default.aspx

§
https://oeps.wv.gov/healthalerts/documents/wv/WVHAN_162.pdf

¶
https://www.wvlegislature.gov/Bill_Text_HTML/2021_SESSIONS/RS/signed_bills/senate/SB334%20SUB1%20ENR_signed.pdf

**
https://library.municode.com/wv/charleston/ordinances/code_of_ordinances?nodeId=1080097

†† Partner services refers to efforts to interview persons with a new diagnosis of HIV and their sexual or needle-sharing partners to offer HIV prevention and treatment and other services. https://www.cdc.gov/hiv/effective-interventions/diagnose/partner-services/index.html

§§ Interview topics for persons who inject drugs included substance use, sexual behavior, barriers to engagement in medical and social services, and strategies to improve engagement in HIV prevention and treatment. Interview topics for community partners included unmet medical and social service needs for persons who inject drugs, barriers to providing HIV and substance use services for persons who inject drugs, and strategies to address the HIV outbreak and improve engagement in HIV prevention and treatment.

¶¶ As of June 18, 2021, 65 persons met the case definition and had one or more health care encounters at sites participating in the medical records abstraction.

*** 45 C.F.R. part 46, 21 C.F.R. part 56; 42 U.S.C. Sect. 241(d); 5 U.S.C. Sect. 552a; 44 U.S.C. Sect. 3501 et seq.

††† Engagement in HIV care was defined as receiving a laboratory test result (CD4, viral load, or genotype test results), documented medical care visit, or antiretroviral treatment prescription in the past 90 days.

§§§ These patients had an HIV-1 viral load test result of <200 HIV RNA copies/mL.

¶¶¶ Opioid-related health care encounters included all encounters in which a person was documented to be using opioids by a clinician, had a positive toxicology screen for opioids, or received syringe services.

****
https://oeps.wv.gov/hiv-aids/documents/data/EpiAid_Report.pdf
